# TRAF6 promotes pulmonary hypertension by enhancing K63-linked ubiquitination and activation of STAT3

**DOI:** 10.3389/fphar.2026.1850163

**Published:** 2026-06-26

**Authors:** Meijing Yao, Rui Chen, Mengran Shen, Zhengqiu Xu, Wei He, Bingbing Li

**Affiliations:** 1 Department of Anesthesiology, Nanjing Drum Tower Hospital, Affiliated Hospital of Medical School, Nanjing University, Nanjing, China; 2 Department of Anesthesiology, Nanjing Drum Tower Hospital Clinical College of Nanjing University of Chinese Medicine, Nanjing, China; 3 Department of Pharmacy, Nanjing Drum Tower Hospital, Affiliated Hospital of Medical School, Nanjing University, Nanjing, China; 4 School of Pharmacy, China Pharmaceutical University, Nanjing, China

**Keywords:** pulmonary artery smooth muscle cell, pulmonary hypertension, pulmonary vascular remodeling, tumor necrosis factor receptor-associated factor 6, ubiquitination

## Abstract

**Background:**

Pulmonary hypertension (PH) is a severe, progressive pulmonary vascular disease characterized by a sustained elevation of pulmonary vascular resistance, *in situ* thrombosis, and progressive vascular remodeling, with limited therapeutic options. In the present study, we aimed to determine whether TRAF6 (tumor necrosis factor receptor-associated factor 6) exacerbates the progression of PH and to elucidate the underlying molecular mechanisms.

**Methods:**

This study examined the protein expression and phosphorylation levels of TRAF6 and signal transducer and transcription activator 3 (STAT3), as well as key biological indicators related to cell proliferation, apoptosis, and migration. By genetic knockdown of TRAF6, we investigated its regulatory role and underlying mechanisms in pulmonary vascular remodeling in both *in vitro* and *in vivo* models of PH.

**Results:**

We employed the monocrotaline (MCT)-induced rat PH model, in which overt pulmonary vascular wall thickening and right ventricular (RV) functional impairment progressively develop. We demonstrate that lung-specific knockdown of TRAF6 markedly attenuates established pulmonary vascular remodeling, improves right ventricular function, and corrects aberrant pulmonary artery smooth muscle cell (PASMC) proliferation, migration, and apoptosis resistance. The molecular interaction between TRAF6 and STAT3 was experimentally confirmed, showing that TRAF6 controls the amount of STAT3 protein and its phosphorylation status by adding ubiquitin to it.

**Conclusion:**

Our study confirmed that TRAF6 plays a key pathogenic role in pulmonary hypertension by promoting STAT3 ubiquitination and sustained activation, thereby accelerating pulmonary vascular remodeling and disease progression. These findings provide a novel mechanistic insight into the progression of PH and highlight TRAF6 as a promising therapeutic target in PH.

## 1 Introduction

Pulmonary hypertension (PH) is a group of severe, progressive pulmonary vascular diseases characterized by elevated pulmonary arterial pressure and pulmonary vascular remodeling (Mocumbi et al., 2024). According to the Lancet Respir Med (Newman and Pepke-Zaba, 2025), an estimated 192,000 cases of pulmonary arterial hypertension were reported worldwide in 2021, with an annual prevalence of 2.28 per 100,000 population and approximately 22,000 associated deaths. Pulmonary vascular remodeling—the central driver of PH onset and progression—is characterized by excessive muscularization of distal pulmonary arteries, resulting in obstructive vascular lesions driven by dysregulated proliferation of endothelial cells (ECs), smooth muscle cells (SMCs), and fibroblasts (Humbert et al., 2019). As a key effector cell type, pulmonary arterial smooth muscle cells (PASMCs) critically contribute to luminal obstruction through enhanced proliferation and migration (Kovacs et al., 2019). Although currently available targeted therapies—including prostacyclin analogs, endothelin receptor antagonists, and phosphodiesterase-5 (PDE-5) inhibitors—improve pulmonary hemodynamics and exercise capacity, none reverse established vascular remodeling or substantially reduce long-term mortality (Coeytaux et al., 2014). Therefore, there remains an urgent unmet need to elucidate the molecular mechanisms driving progressive pulmonary arterial narrowing and to develop novel therapies capable of reversing vascular remodeling.

Tumor necrosis factor receptor-associated factor 6 (TRAF6) is the only TRAF family member that participates in both the TNFR superfamily and the interleukin-1 receptor/Toll-like receptor (IL-1R/TLR) superfamily signaling pathways, positioning it at a central node in innate immunity, inflammation, cell proliferation, and survival. In hypertensive mice, pharmacological inhibition of TRAF6 reduces immune cell recruitment to the aortic wall, restores endothelial function, and lowers blood pressure (Strohm et al., 2025). TRAF6 silencing also attenuates endothelial pyroptosis in atherosclerosis (Zhang Y. et al., 2023) and suppresses vascular smooth muscle cell calcification, thereby reducing plaque burden (Yang et al., 2022). These aforementioned studies underscore the multifaceted roles of TRAF6 in vascular pathology. However, its specific mechanisms in PH remain insufficiently explored. To date, Wang et al. (2024) have reported that sevoflurane inhibits PASMC phenotypic switching and NF-κB pathway activation through downregulation of TRAF6 expression. Additionally, another study demonstrated that in a hypoxia-induced mouse model of PH, TRAF6 promotes mitophagy, proliferation, and migration in human pulmonary artery smooth muscle cells (HPASMCs) via the inositol-requiring enzyme 1/X-box binding protein 1 (IRE1/XBP1) axis (Wang et al., 2026). Although these studies confirm that TRAF6 can modulate PASMC functions, whether it constitutes a critical pathogenic driver of PH requires further experimental validation.

As an E3 ubiquitin ligase, TRAF6 determines the fate of substrate proteins by catalyzing distinct ubiquitin chain linkages. Ubiquitination, which covalently attaches the C-terminal glycine of ubiquitin to the ε-amino group of lysine residues on substrate proteins, broadly regulates cellular processes (Swatek and Komander, 2016). Notably, the regulation of signal transducer and activator of transcription 3 (STAT3) proteins by TRAF6 has been validated in other disease contexts. Li et al. (2020) reported that GAS5 promotes TRAF6-mediated ubiquitination and degradation of STAT3, thereby suppressing Th17 differentiation and ameliorating immune thrombocytopenia. Meanwhile, miR-125b enhances p-JAK2 and p-STAT3 expression through negative regulation of TRAF6, thereby exacerbating the progression of osteoporosis (Wang et al., 2021). These findings suggest that the TRAF6-STAT3 axis may serve as a central regulatory node across diverse pathological processes.

In addition, STAT3 functions as a critical nuclear transcription factor that broadly governs cell growth, apoptosis, and immune responses (Liu et al., 2021). Within the context of pulmonary hypertension (PH), excessive STAT3 activation has been firmly established as a major driving force behind pulmonary vascular remodeling. Specifically, under hypoxic conditions, the Ntsr1-JAK2-STAT3-Thbs1 pathway has been shown to augment endoplasmic reticulum (ER) stress via activating transcription factor 6 (ATF6) activation, thereby enhancing PASMC proliferative and migratory capacities (Wei et al., 2024). In parallel, in monocrotaline (MCT)-induced PH rats, phosphorylation-mediated STAT3 activation upregulates SHP2 expression, which in turn promotes PASMC proliferation and confers resistance to apoptosis (Courboulin et al., 2011). Similarly, in chronic hypoxia-induced PH models, STAT3 directly binds to the CCNA promoter to facilitate Cyclin A2 transcription, actively driving vascular remodeling (Zhang et al., 2020). However, comprehensive understanding of whether and how TRAF6 regulates STAT3 to exacerbate PH progression remains limited. Moreover, the specific ubiquitin-mediated modulation of STAT3 in the MCT-induced PH model has yet to be elucidated. Accordingly, this study aimed to investigate the regulatory interplay between TRAF6 and STAT3 in PASMCs and in a rat model of MCT-induced PH, to define the significance of the TRAF6-STAT3 axis in pulmonary vascular remodeling, to provide novel mechanistic insights into PH pathogenesis, and to establish a theoretical foundation for subsequent therapeutic targeting of TRAF6.

## 2 Materials and methods

### 2.1 Animals

Male Sprague-Dawley (SD) rats weighing 180–200 g (6 weeks old) were purchased from SPF Biotechnology Co., Ltd. (Beijing, China). The animals were housed at the Nanjing First Hospital Laboratory Animal Center under designated pathogen-free conditions and controlled temperature of 20 °C–26 °C and relative humidity of 40%–70%. Each animal experiment was conducted in accordance with standards established by the Nanjing First Hospital Ethics Committee (Approval No. DWSY-24153611).

### 2.2 Adeno-associated virus vector (AAV) construction

Recombinant single-stranded AAV vectors expressing short hairpin RNA targeting rat TRAF6 (AAV1-shRNA-TRAF6) or scrambled control (AAV1-shRNA-NC) under the control of the SM22α promoter, together with an EGFP reporter (ssAAV-SM22α-EGFP backbone), were designed and packaged into serotype AAV1 by PackGene Biotech Co., Ltd. (Guangzhou, China). Viral particles were purified, and the final titer was 1 × 10^13^ viral genomes (vg)/mL. For detailed information on AAV, please refer to Supplementary Table S4 in the attachment.

### 2.3 Animal model establishment and experimental grouping

A total of 36 male Sprague-Dawley rats were used in the first part of this study. Among them, nine rats were assigned to the control group, and the remaining 27 rats received a single subcutaneous injection of 60 mg/kg MCT (Sigma-Aldrich, St. Louis, MO, USA) in the dorsal neck region for time-course grouping. A separate cohort of 30 rats was randomly divided into four groups: Control (n = 6), MCT (n = 8), MCT + shRNA-NC (n = 8), and MCT + shRNA-TRAF6 (n = 8). The PH model was induced by a single subcutaneous injection of 60 mg/kg MCT, while the control group received an equivalent volume of saline. One week after MCT administration, rats in the MCT + shRNA-NC and MCT + shRNA-TRAF6 groups were anesthetized with pentobarbital sodium (50 mg/kg, intraperitoneal injection). Subsequently, AAV1-shRNA-TRAF6 and its negative control AAV1-shRNA-NC were delivered via a single intratracheal instillation at a titer of 1 × 10^12^ vg/mL (Jiao et al., 2025).

### 2.4 Isolation and culture of rat PASMCs

Immediately after anesthetizing and euthanizing rats, the heart and lung tissues were removed. Under a microscope in a sterile workbench, the pulmonary artery was dissected. Using microforceps, the adventitia and endothelium were removed, leaving only the media. This media tissue was cut into approximately 1 mm^3^ fragments and uniformly seeded into 25 cm^2^ culture flasks. After incubating in the incubator for 6 h, culture medium was added. Cell growth was observed after 4–6 days, and the culture was passaged for subsequent experiments. See Supplementary Figure S1 for the characterization of primary PASMCs.

### 2.5 siRNA and plasmid transfection

siRNAs targeting rat TRAF6 (siTRAF6) and nontargeting control siRNA (siNC) were obtained from Sangon Biotech (Shanghai, China). Sequences are provided in Supplementary Table S2. The pcDNA3.1-3 × FLAG-TRAF6 plasmid was synthesized by YouBio Biological Technology (Changsha, China). HA-ubiquitin (HA-Ub) wild-type and mutants (K48-only, K48R, K63-only, K63R) and FLAG-STAT3 constructs were generated by PCR amplification and subcloned into pcDNA3.1 vectors using EcoRI and XhoI restriction sites. All constructs were verified by Sanger sequencing. Plasmids were amplified in DH5α competent cells and purified using an endotoxin-free plasmid maxi kit (Qiagen). Primary PASMCs were seeded in 6- or 12-well plates and grown to 70% confluence in DMEM supplemented with 10% FBS and 1% penicillin–streptomycin. Cells were transfected with siRNA (50 nM) or plasmid DNA (3 μg/well in 6-well plates) using Lipofectamine 3000 (Thermo Fisher Scientific) according to the manufacturer’s instructions. Experiments were performed 48 h (protein) or 24–48 h (mRNA) after transfection.

### 2.6 Echocardiography

Four weeks after MCT or saline injection, rats were anesthetized with 2%–3% isoflurane and examined using small-animal ultrasound system (VINNO 6VET). Left-ventricular long-axis M-mode recordings were obtained to measure stroke volume (SV) and ejection fraction (EF) for assessment of left-sided systolic and diastolic function. Pulsed-wave Doppler of the right-ventricular outflow tract (RVOT) was performed from the parasternal short-axis view to determine pulmonary artery acceleration time (PAAT, ms) and pulmonary ejection time (PET, ms). Tricuspid annular plane systolic excursion (TAPSE, mm) was measured by M-mode in the apical four-chamber view to evaluate right-ventricular function.

### 2.7 Histopathology and immunohistochemistry

Left upper lung lobes were fixed in 4% neutral buffered formalin for 24 h, paraffin-embedded, and cut into 4 μm sections. Hematoxylin-eosin (H&E) staining was performed using standard protocols. For immunohistochemistry, sections were subjected to antigen retrieval in citrate buffer (pH 6.0) at 98 °C for 20 min, blocked with 3% hydrogen peroxide and 5% normal goat serum, and incubated overnight at 4 °C with anti-Ki67 antibody. HRP-conjugated secondary antibody was applied, followed by DAB development and hematoxylin counterstaining. Negative controls omitted the primary antibody.

### 2.8 Cell proliferation, migration and apoptosis assays

Pre-treated PASMCs were seeded at 3,000 cells per well in 96-well plates and cultured in DMEM containing 5% FBS. After 4 h adhesion (defined as 0 h), 10 µL CCK-8 reagent was added at 24, 48 and 72 h; absorbance at 450 nm was recorded. PASMCs in 6-well plates were subjected to EdU labeling (Beyotime, Shanghai, China), fixation, permeabilization, and DAPI counterstaining. EdU-positive cells were quantified using ImageJ. Migration was evaluated by scratch-wound assay using Culture-Insert 2 Well. Images were captured at 0, 6, 12, and 30 h; wound area was quantified using ImageJ. Apoptosis was detected using the One Step TUNEL Apoptosis Assay Kit (Beyotime Biotechnology, Shanghai, China) according to the manufacturer’s protocol.

### 2.9 Protein extraction and immunoblotting

Tissues or cells were ice-cold RIPA lysis buffer supplemented with protease and phosphatase inhibitors. After centrifugation at 12,000 × g for 15 min at 4 °C, supernatants were collected and quantified by BCA assay. Equal amounts of protein (25 μg) were separated by 6%–12% SDS-PAGE, transferred to PVDF membranes, blocked for 1 h at room temperature with 5% non-fat milk in TBST, and probed overnight at 4 °C with primary antibodies against STAT3, p-STAT3, TRAF6, cleaved caspase3, HA-tag, FLAG-tag, and β-actin (all antibodies are detailed in Supplementary Table S1). After three washes (10 min each) with TBST, membranes were incubated with HRP-conjugated secondary antibodies for 1 h at room temperature. Immunoreactive bands were visualized using enhanced chemiluminescence reagent and imaged on a ChemiDoc imaging system. Band intensities were quantified using ImageJ and normalized to β-actin.

### 2.10 Co-immunoprecipitation (Co-IP)

Cells were lysed in ice-cold NP-40 lysis buffer (50 mM Tris-HCl pH 7.4, 150 mM NaCl, 1% NP-40, 1 mM EDTA) supplemented with protease and phosphatase inhibitors. Protein concentration was determined by BCA assay. For each reaction, 500–1,000 μg of pre-cleared lysate was incubated with 2–4 μg of anti-STAT3 antibody overnight at 4 °C on a rotator. Pre-washed Protein A/G magnetic beads (40 μL) were added and incubated for an additional 2 h at 4 °C. Beads were washed four times with 1 mL ice-cold lysis buffer, resuspended in 2× SDS loading buffer, and boiled for 10 min. Input samples (5% of total lysate) were processed in parallel. Immunoprecipitated proteins were resolved by SDS-PAGE and analyzed by Western blotting.

### 2.11 Detection of protein ubiquitination

PASMCs were co-transfected with HA-tagged ubiquitin (HA-Ub) constructs and the indicated FLAG-tagged plasmids using Lipofectamine 3000. Forty-eight hours after transfection, cells were treated with 10 μm MG132 for 6 h and lysed in ice-cold NP-40 lysis buffer (50 mM Tris-HCl pH 7.4, 150 mM NaCl, 1% NP-40, 1 mM EDTA) supplemented with protease/phosphatase inhibitors and 10 mM N-ethylmaleimide (NEM). Lysates containing 800 μg total protein were pre-cleared with 20 μL Protein A/G magnetic beads for 30 min at 4 °C. STAT3 was immunoprecipitated overnight at 4 °C with 2 μg anti-STAT3 antibody or normal rabbit IgG (isotype control). Pre-washed Protein A/G magnetic beads (40 μL) were added and incubated for an additional 2 h at 4 °C. Beads were washed four times with 1 mL ice-cold lysis buffer, resuspended in 2× SDS loading buffer containing 100 mM DTT, and boiled for 10 min. Input (5%) and immunoprecipitates were analyzed by Western blotting with anti-HA (for ubiquitin) or anti-FLAG antibodies.

### 2.12 Total RNA isolation and real-time quantitative PCR

Total RNA was extracted from lung tissues or cultured PASMCs using TRIzol Reagent (Invitrogen) according to the manufacturer’s protocol. RNA concentration and purity (A260/A280 = 1.9–2.1) were measured using spectrophotometer. First-strand cDNA was synthesized from 500 ng total RNA using HiScript III Reverse Transcriptase. RT-qPCR was performed in triplicate in 20 μL reactions containing 2 μL cDNA, 10 μL SYBR qPCR Master Mix, 0.4 μL each primer (10 μM), and 7.2 μL nuclease-free water on a QuantStudio 6 Flex System. Cycling conditions were: 95 °C for 30 s, followed by 40 cycles of 95 °C for 5 s and 60 °C for 30 s, and a melting curve from 65 °C to 95 °C. Relative mRNA expression was calculated using the 2^−ΔΔCT^ method and normalized to Actb. Primer sequences are listed in Supplementary Table S3.

### 2.13 Immunofluorescence staining

For cell imaging, PASMCs cultured on glass coverslips were fixed with 4% paraformaldehyde for 15 min at room temperature (RT), washed three times with PBS, permeabilized with 0.1% Triton X-100 for 10 min and blocked with 5% bovine serum albumin (BSA) for 1 h at RT. Coverslips were then incubated overnight at 4 °C with primary antibodies against STAT3, TRAF6 and p-STAT3. After three PBS washes, appropriate Alexa Fluor-conjugated secondary antibodies were applied for 1 h at RT in the dark. For α-smooth muscle actin (α-SMA) visualization, FITC-conjugated anti-α-SMA antibody was used directly. Nuclei were counterstained with DAPI in antifade mounting medium. For lung tissue, fresh specimens were dehydrated, embedded and sectioned at 20 µm by Servicebio (Wuhan, China). The staining protocol was identical to that described for cells. Images were acquired using a LACA fluorescence microscope (Leica, Germany).

### 2.14 Statistical analysis

Data were analyzed using GraphPad Prism version 9.0. Normality and equal variance were assessed using the Shapiro-Wilk test and Brown-Forsythe/Bartlett’s tests, respectively. Normally distributed data were compared using an unpaired, two-tailed Student’s t-test (two groups) or one-way or two-way ANOVA followed by Šídák’s or Dunnett’s multiple-comparison test (multiple groups). Data are presented as mean ± SEM from at least three independent experiments. *P* < 0.05 was considered statistically significant (^*^ *P* < 0.05, ^**^ *P* < 0.01, ^***^ *P* < 0.001, ^****^ *P* < 0.0001).

## 3 Results

### 3.1 Elevated TRAF6 expression in lung tissues of MCT-PH rats

**FIGURE 1:**
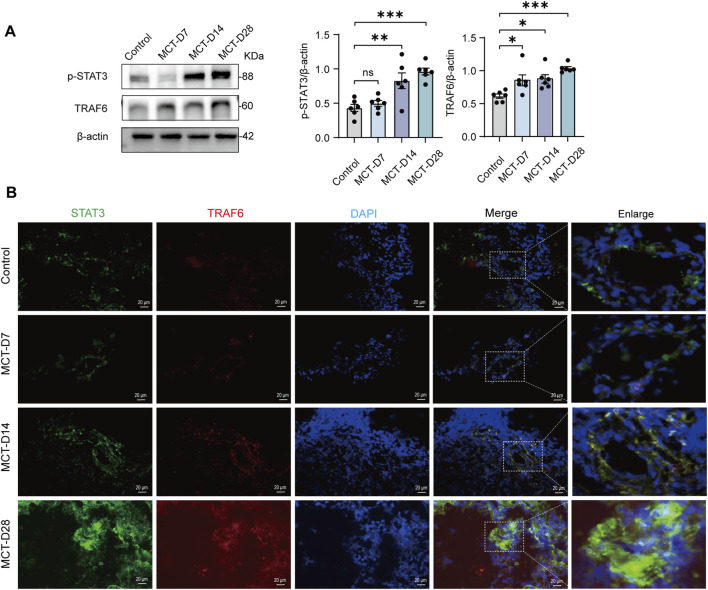
Elevated levels of TRAF6, STAT3 and p-STAT3 in lung tissues from MCT-induced rats. **(A)** Representative Western blotting analyses of TRAF6, p-STAT3 expression in the lung tissues of MCT model rat, n = 6. **(B)** Immunofluorescence staining images of TRAF6 and STAT3 in lung tissues from MCT rats at different time points post-modeling. Frozen sections were stained for TRAF6 (red), STAT3 (green), and DAPI (blue). Scale bar = 20 μm. All the data are presented as mean ± SEM. Compared with the control group: *P < 0.05, **P < 0.01, ***P < 0.001; ns indicates no significant difference (*P* ≥ 0.05).

To determine TRAF6 expression in lung tissues from control and MCT rats, 9 of the 36 rats were designated as controls, and the remaining 27 MCT-treated rats were randomly divided into three groups (n = 9 per group). Rats were euthanized at 7, 14, and 28 days post-modeling, respectively, and lung tissues were collected for Western blot analysis. The results showed that compared with the control group, the protein expression levels of TRAF6 and p-STAT3 were significantly increased in a time-dependent manner (Figure 1A). In addition, immunofluorescence staining of lung tissues revealed that both TRAF6 and STAT3 were upregulated in the perivascular regions under disease conditions, as evidenced by stronger fluorescence signals observed at 14 and 28 days post-modeling (Figure 1B). In summary, our data demonstrate that TRAF6 and STAT3 are upregulated in PH in a time-dependent manner following disease induction, and may contribute to PH pathogenesis.

### 3.2 Expression and distribution of TRAF6 and STAT3 in pulmonary vasculature and PASMCs

**FIGURE 2:**
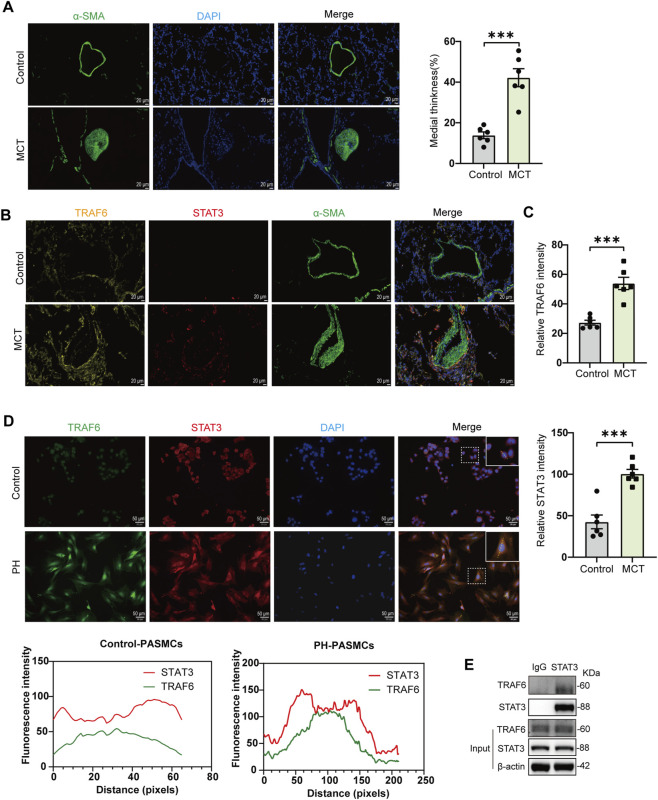
Co-expression of TRAF6 and STAT3. **(A)** Representative images and quantitative analysis of α-SMA immunofluorescence staining in paraffin-embedded sections. Scale bar = 20 µm. **(B, C)** Representative immunofluorescence staining images and quantification TRAF6 and STAT3 in lung tissues from control and MCT rats. Paraffin sections were stained for TRAF6 (yellow), STAT3 (red), α-SMA (green), and DAPI (blue). Scale bar = 20 μm. **(D)** Representative immunofluorescence images of TRAF6 and STAT3 and colocalization analysis in control-PASMCs and PH-PASMCs. Cells grown on coverslips were stained for TRAF6 (green), STAT3 (green), and DAPI (blue). Scale bar = 50 μm. **(E)** Co-IP of STAT3 in PASMCs from MCT-PH rats. Immunoprecipitates and 5% input were immunoblotted with the indicated antibodies.

First, α-SMA served as a medial layer marker to reflect the thickness of the pulmonary arterial media. Immunofluorescence single staining results showed that the medial layer of the pulmonary vascular wall in the MCT group was thickened compared with the control group (Figure 2A). Meanwhile, to investigate the potential association between TRAF6 and STAT3, immunofluorescence staining was performed on lung tissues from both control and MCT group rats. The results indicated that both molecules were upregulated in PH disease tissues, showing diffuse distribution throughout the lung tissue with partial overlap of their fluorescent signals observed around the blood vessels (Figures 2B,C). Subsequently, immunofluorescence staining was conducted on pulmonary artery smooth muscle cells. The results suggested that TRAF6 and STAT3 were upregulated in PH-PASMCs, with overlapping fluorescent signals (Figure 2D). These observations prompted us to examine whether a physical interaction exists between these proteins. Co-IP assay confirmed that TRAF6 interacts with STAT3 (Figure 2E). Collectively, these findings demonstrate that TRAF6 interacts with STAT3 and may be functionally correlated in the pathogenesis of PH.

### 3.3 TRAF6 regulates the PASMC phenotype

**FIGURE 3:**
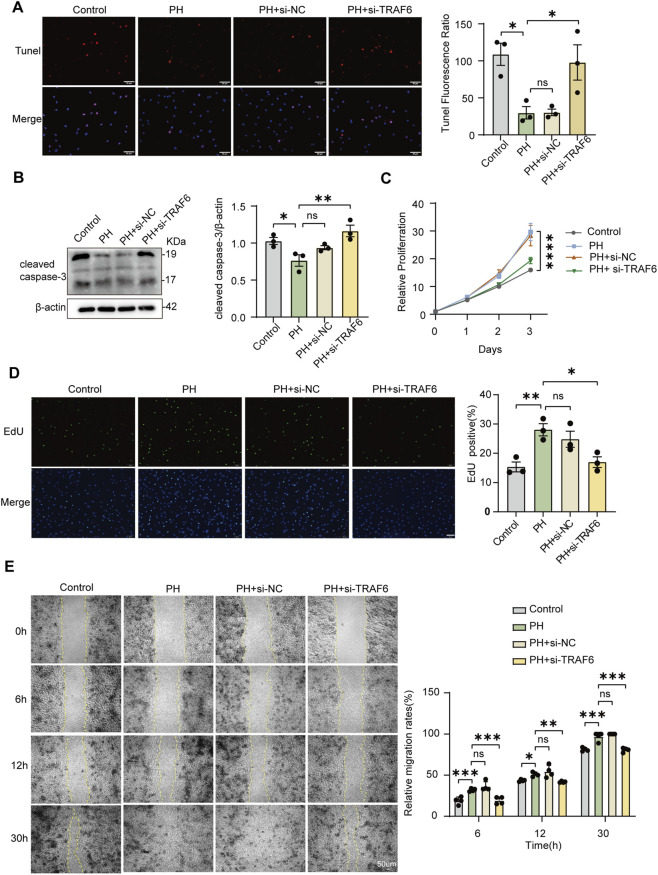
si-TRAF6 ameliorates proliferation, apoptosis resistance, and migration of primary rat PASMCs. **(A)** Representative TUNEL staining in PASMCs. Total nuclei were counterstained with DAPI (blue). Scale bar = 50 μm. **(B)** Western blotting of cleaved caspase-3 and β-actin in PASMCs transfected with control or TRAF6 siRNA. **(C)** CCK-8 proliferation assay of PASMCs transfected with control or TRAF6 siRNA. Absorbance at 450 nm was measured at 0, 1, 2, and 3 days after seeding 3,000 cells/well. **(D)** Representative fluorescence images and quantitative analysis of EdU-positive cells; blue fluorescence indicates nuclear staining (DAPI). Scale bar = 100 μm. **(E)** Scratch-wound migration assay of PASMCs transfected with control or TRAF6 siRNA. Images were acquired at 0, 6, 12 and 30 h after scratch. Migration distance was quantified using ImageJ. Scale bar = 50 μm. Data are mean ± SEM from n = 3 independent experiments. *P < 0.05, **P < 0.01, ***P < 0.001, ****P < 0.0001, ns (not significant) vs. PH; one-way ANOVA with Dunnett’s multiple-comparison test **(A–D)** or two-way ANOVA with Šídák’s multiple-comparison test **(E)**.

Focusing on PASMCs—the principal cellular component of the arterial media—we next asked whether TRAF6 governs the pathological proliferative, apoptotic and migratory phenotype that underlies medial thickening and muscularization in PH. TUNEL staining showed that PH-PASMCs exhibited marked apoptosis resistance compared with control cells; this phenotype was reversed by TRAF6 silencing (Figure 3A), a finding corroborated by WB analysis of cleaved caspase-3 (Figure 3B). Concurrently, cell proliferation was detected by CCK-8 assay combined with EdU staining. The results showed that the proliferative capacity of PH-PASMCs was enhanced, which was suppressed after TRAF6 knockdown (Figures 3C,D). In scratch-wound assays, TRAF6 knockdown significantly impaired the accelerated migration of PH-PASMCs, leaving visible gaps at 30 h, whereas control siRNA-transfected cells had achieved complete wound closure (Figure 3E).

Overall, these findings demonstrate that TRAF6 orchestrates the proliferative, antiapoptotic and promigratory profile of PASMCs, indicating that it plays a central role in the pathological process of PH.

### 3.4 TRAF6 modulates STAT3 ubiquitination and phosphorylation

**FIGURE 4:**
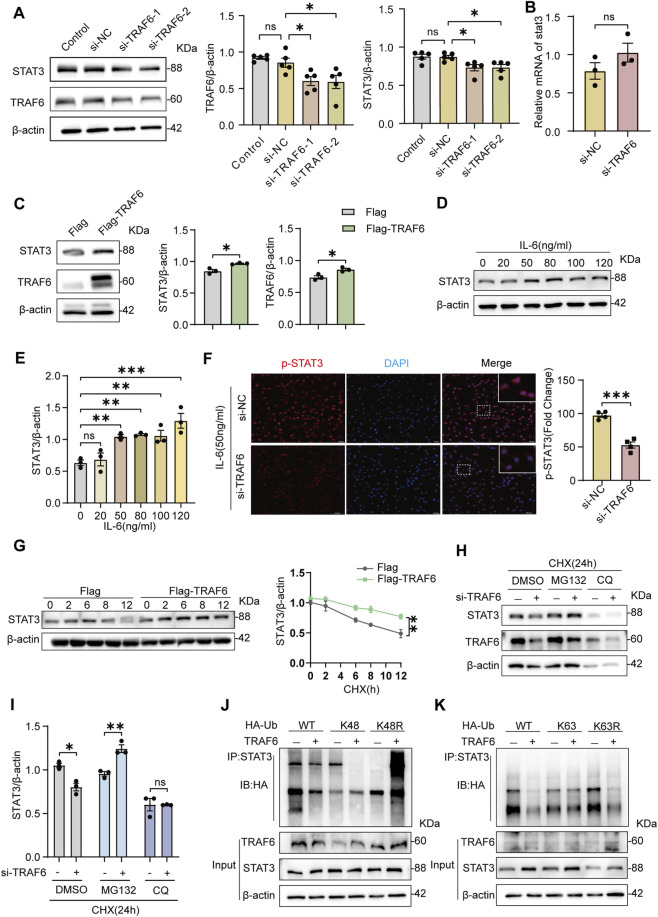
TRAF6 overexpression or knockdown modulates STAT3 protein stability, phosphorylation, and ubiquitin chain topology in PASMCs. **(A, B)** PASMCs were transfected with control or TRAF6 siRNA for 24 h (mRNA) or 48 h (protein). STAT3 mRNA was quantified by RT-qPCR using the 2^−ΔΔCT^ method (n = 3); protein levels of TRAF6 and STAT3were determined by Western blotting and densitometric analysis using ImageJ (normalized to β-actin, n = 5). **(C)** PASMCs were transfected with empty vector or FLAG-TRAF6 for 48 h; STAT3 protein was assessed by Western blotting. **(D, E)** Dose-dependent STAT3 in response to 0–100 ng/mL IL-6 for 30 min **(F)** PASMCs transfected with control or TRAF6 siRNA for 24 h were stimulated with 50 ng/mL IL-6 for 30 min, fixed, and immunostained for p-STAT3 (red) with DAPI counterstain (blue). Nuclear p-STAT3 fluorescence intensity was quantified using ImageJ. Scale bar = 50 μm. **(G–I)** Cycloheximide (CHX) chase and proteasome/autophagy inhibition assays assessing STAT3 protein half-life (detailed in methods). **(J, K)** Ubiquitin chain topology analysis: PASMCs were co-transfected with HA-ubiquitin (WT, K48-only, K48R, K63-only, or K63R) ± FLAG-TRAF6. STAT3 was immunoprecipitated and immunoblotted for HA to detect linkage-specific ubiquitination. Data are mean ± SEM from n = 3 independent experiments. *P < 0.05, **P < 0.01, ***P < 0.001 vs. respective control; unpaired, two-tailed Student’s t-test **(B, C, F)**, one-way ANOVA followed by Dunnett’s multiple-comparison test **(A, D, E)**, or two-way ANOVA followed by Šídák’s multiple-comparison test **(G–I)**.

Building on our co-IP data confirming a TRAF6–STAT3 interaction (Figure 2E), we next asked whether TRAF6 regulates STAT3 protein stability or activity. PASMCs were transfected with TRAF6 siRNA or FLAG-TRAF6 plasmid. Western blotting showed that STAT3 protein levels decreased upon TRAF6 knockdown and increased following TRAF6 overexpression (Figures 4A,C), whereas RT-qPCR revealed no change in STAT3 mRNA (Figure 4B), implying a post-translational mechanism. To test this, we assessed STAT3 protein half-life after cycloheximide-mediated translational inhibition (Miao et al., 2023). STAT3 decayed markedly more slowly in FLAG-TRAF6-overexpressing cells (Figure 4G), indicating that TRAF6 protects STAT3 from degradation. Proteasome inhibition with MG132 (but not autophagy inhibition with chloroquine) abolished STAT3 loss and nullified the accelerating effect of TRAF6 knockdown (Figures 4H,I), pinpointing the proteasome as the relevant degradation pathway.

We then examined STAT3 functional activation. Stimulation with IL-6 (50 ng/mL) was used to trigger JAK/STAT signaling (Figures 4D,E) (Johnson et al., 2018; Li et al., 2017). Immunofluorescence revealed that TRAF6 silencing curtailed both STAT3 phosphorylation and nuclear translocation (Figure 4F), indicating that TRAF6 not only stabilizes STAT3 but also facilitates its activation.

Finally, we interrogated ubiquitin linkage specificity. PASMCs were co-transfected with HA-ubiquitin mutants (WT, K48-only, K48R, K63-only, K63R) and FLAG-TRAF6. TRAF6 overexpression reduced K48-linked ubiquitination while enhancing K63-linked ubiquitination of STAT3 (Figures 4J,K). K48-linked chains target proteins for proteasomal degradation, whereas K63-linked chains serve as non-degradative scaffolds that promote kinase activation and signaling. These data indicate that TRAF6 switches the ubiquitin code of STAT3 from degradative (K48) to activating (K63), thereby simultaneously preventing proteasomal degradation and promoting phosphorylation-dependent activation.

### 3.5 *In vivo* knockdown of TRAF6 attenuates MCT-induced pulmonary hypertension in rats

**FIGURE 5:**
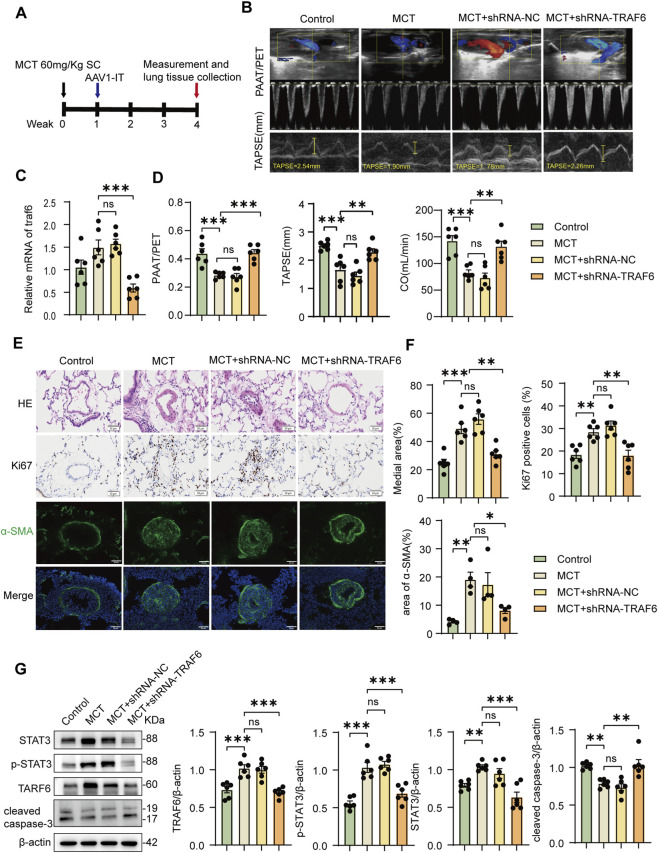
AAV1-shTRAF6 ameliorates right ventricular dysfunction and pulmonary arterial wall thickening in MCT-induced PH rats. **(A)** Experimental timeline. Male rats received a single subcutaneous injection of MCT (60 mg/kg) or saline on day 0. One week later, AAV1-shTRAF6 or AAV1-shNC (1 × 10^12^ vg/rat) was delivered by intratracheal instillation. Echocardiography and tissue collection were performed 4 weeks after MCT injection. **(B)** Representative pulsed-wave Doppler (PAAT/PET) and M-mode (TAPSE) echocardiographic images. **(C)** RT-qPCR analysis of pulmonary TRAF6 mRNA expression (n = 6). **(D)** Quantification of PAAT/PET, TAPSE, and cardiac output (CO) (n = 6). **(E, F)** Representative hematoxylin-eosin (H&E) staining, Ki67 immunohistochemistry, and α-SMA (green) immunofluorescence with DAPI counterstain (blue) of lung sections. Medial wall thickness, percentage of Ki67^+^cells, and α-SMA intensity were quantified. Scale bars = 50 μm. **(G, H)** Western blot analysis and quantification of TRAF6, STAT3, p-STAT3, and cleaved caspase-3 in lung homogenates (normalized to β-actin; n = 6). Data are mean ± SEM from n = 6 rats per group. *P < 0.05, **P < 0.01, ***P < 0.001 vs. MCT; ns, not significant; one-way ANOVA with Dunnett’s multiple-comparison test.

To determine the *in vivo* impact of TRAF6 on PH progression, we delivered AAV1 vectors encoding TRAF6 shRNA (AAV1-shTRAF6) or scrambled control (AAV1-shNC) to the lung tissue of adult rats via intratracheal instillation (Figure 5A). Three weeks after viral administration, both TRAF6 mRNA and protein levels in whole-lung lysates were significantly reduced (Figures 5C,G), confirming lung-directed knockdown.

Echocardiographic assessment was performed to evaluate pulmonary hemodynamics and right ventricular function. Compared with saline control rats, MCT-treated rats exhibited significantly decreased PAAT/PET ratio, reduced TAPSE, and lower cardiac output (CO), indicating increased pulmonary vascular resistance and impaired right ventricular systolic function. These echocardiographic abnormalities were significantly attenuated in the MCT + AAV1-shTRAF6 group, with partial restoration of PAAT/PET and TAPSE, as well as improved CO (Figures 5B,D). Medial thickening is a key hallmark of pulmonary hypertension severity. Hematoxylin and eosin (H&E) staining and α-SMA immunofluorescence showed prominent medial hyper-trophy in the pulmonary arteries of MCT rats, which was significantly attenuated by TRAF6 knockdown (Figures 5E,F). In addition, Ki67 immunostaining revealed a significantly increased percentage of Ki67-positive cells in MCT lungs, which was markedly reduced following TRAF6 silencing. Western blotting of fresh lung tissue confirmed efficient knockdown of TRAF6 and showed concomitant decreases in STAT3 and p-STAT3 protein levels, whereas cleaved caspase-3 was increased (Figure 5G). Together, these findings demonstrate that *in vivo* TRAF6 inhibition suppresses STAT3 signaling and downstream antiapoptotic pathways, thereby inducing structural alterations in the pulmonary arteries, with subsequent luminal narrowing further driving the progression of PH.

## 4 Discussion

PH is a progressive and fatal vascular disorder for which no curative treatment currently exists; this therapeutic limitation is closely tied to the incomplete understanding of its primary pathogenic drivers (Bousseau et al., 2023). In the present study, we demonstrate that TRAF6 modulates STAT3 phosphorylation by enhancing K63-linked ubiquitination of STAT3 in PASMCs, thereby influencing PASMC proliferation, apoptosis resistance, and migratory phenotype, and ultimately affecting the onset and progression of PH in rats.

Pulmonary vascular remodeling, characterized by aberrant proliferation and phenotypic switching of PASMCs, represents the hallmark pathological feature of PH (Humbert et al., 2023). Recently, TRAF6 has emerged as a critical regulator of inflammation and cell survival in cardiovascular diseases. Chouvarine et al. reported significant TRAF6 upregulation in myocardial tissue from end-stage PH patients (Chouvarine et al., 2019), while Song et al. and Dong et al. demonstrated that TRAF6 mediates NF-κB activation, monocyte infiltration, and vascular smooth muscle cell survival during vascular remodeling and post-injury repair (Song et al., 2012; Dong et al., 2015). However, the expression profile of TRAF6 in PH pulmonary vasculature and its specific role in PASMCs remain poorly understood.

In the present study, we revealed that both TRAF6 and STAT3 were coordinately upregulated in PH lung tissues and PASMCs, with direct physical interaction between them. Mechanistically, TRAF6 stabilized STAT3 protein and facilitated its phosphorylation-dependent activation, thereby promoting PASMC proliferation, migration, and apoptosis resistance. *In vivo*, TRAF6 knockdown partially ameliorated right ventricular dysfunction and pulmonary vascular wall thickening in the MCT-induced PH rat model. These findings identify the TRAF6-STAT3 axis as a critical mechanism driving the pathological phenotypic transformation of PASMCs.

Consistent with our observations, TRAF6 upregulation has been documented in various vascular diseases and positively correlates with disease severity, including renal injury and aortic inflammation (Strohm et al., 2025), hypertension-related vascular inflammation (He et al., 2024; Lameijer et al., 2018), and hypoxia-induced PASMC phenotypic switching via inflammatory cytokine activation (Adu-Amankwaah et al., 2025). Notably, Luo et al. further demonstrated that TRAF6 promotes right ventricular remodeling through K63-linked ubiquitination of NF-κB p65 and downstream MAPK activation in the MCT-PH model (Luo et al., 2024). Collectively, these studies support the therapeutic potential of targeting TRAF6 in PH.

STAT3 serves as a canonical transcription factor that regulates proliferation, survival, and angiogenesis across diverse pathological contexts (Hu et al., 2024). In the pulmonary vasculature, the DYRK1A/STAT3/Pim-1/NFAT axis enhances PASMC proliferation and migration while suppressing apoptosis, closely correlating with PAH-associated remodeling (Lan et al., 2024). Additionally, augmented Hsp110–STAT3 interaction elevates p-STAT3 and c-Myc levels in pulmonary arterial endothelial cells, promoting aberrant proliferation and migration (Zhao et al., 2023). These findings establish STAT3 activation as a convergent mechanism driving vascular cell dysfunction in PH.In our study, we demonstrated that TRAF6 knockdown markedly suppressed both total and phosphorylated STAT3 protein levels in PASMCs, revealing a positive regulatory relationship whereby TRAF6 promotes STAT3 phosphorylation. Mechanistically, TRAF6 protected STAT3 from proteasomal degradation and enhanced its transcriptional activity, identifying a post-translational regulatory node upstream of STAT3 signaling.

The pro-angiogenic and pro-proliferative functions of STAT3 are well documented in vascular remodeling. STAT3-driven glycolytic reprogramming fuels excessive cellular proliferation (Zhang C. et al., 2023), while endothelial-specific STAT3 deletion reduces tumor vascular density (Shen et al., 2021) and pharmacological STAT3 inhibition attenuates pulmonary artery pressure and vascular remodeling (Bisserier et al., 2021). Clinically, STAT3-targeted therapies for vascular diseases are rapidly evolving (Dutzmann et al., 2015). Our findings that TRAF6 mediates K63-linked ubiquitination to stabilize and activate STAT3 not only elucidate a novel upstream regulatory mechanism but also position the TRAF6–STAT3 axis as a potential therapeutic target for PH intervention.

Ubiquitination is a crucial post-translational modification. K48-linked ubiquitin chains typically target substrates for proteasomal degradation (Manohar et al., 2019), whereas K63-linked chains primarily serve as non-degradative scaffolds that facilitate protein–protein interactions and kinase activation (Cao et al., 2022). In the present study, we observed elevated K63-linked ubiquitination of STAT3 accompanied by a decrease in K48-linked ubiquitination. The paradoxical reduction in total STAT3 ubiquitination despite elevated K63-linked chains may be attributable to the formation of branched or mixed ubiquitin chains (Ohtake et al., 2016) and selective activation of K63-preferring E3 ligases coupled with differential inhibition of specific deubiquitinases (DUBs) (Waltho et al., 2024). Although these mechanisms may partially account for our observations, additional mechanistic studies will be required to fully clarify the regulation of STAT3 ubiquitin chain topology in pulmonary hypertension.

Despite comprehensive experimental validation, several limitations should be noted. First, while the MCT model effectively induces pulmonary vascular remodeling, complementary models such as hypoxia, SU5416/hypoxia (SuHx), and high pulmonary blood flow better recapitulate the multifactorial nature of human PH. Consequently, the generalizability of our TRAF6-STAT3 findings remains to be tested in these alternative systems. Second, invasive hemodynamic measurements (e.g., RVSP and mPAP) and the Fulton index RV/(LV+S) were not assessed in the present study. We evaluated pulmonary hemodynamics and right ventricular function primarily through non-invasive echocardiography (PAAT/PET ratio and TAPSE). Although these parameters have been validated in previous MCT-PH studies, the absence of direct invasive and histopathological data may somewhat limit the precision of our conclusions regarding disease severity and right ventricular remodeling. Third, species-specific differences between rats and humans may limit direct clinical translational potentia. Finally, we primarily examined K48- and K63-linked ubiquitination using linkage-specific ubiquitin mutants. However, STAT3 may also be modified by less common linkages (K6-, K11-, K27-, and K29-linked), as well as mixed or linear chains (Cho et al., 2019; Cockram et al., 2021), whose abundance and functional roles under pro-inflammatory conditions remain to be fully elucidated.

In summary, TRAF6 knockdown ameliorates pulmonary vascular remodeling in PH by suppressing K63-linked ubiquitination and subsequent phosphorylation of STAT3, thereby restoring normal PASMC function. Our findings identify TRAF6 as a highly promising therapeutic target for PH and provide important mechanistic insight into its critical role in medial thickening. Although our data link TRAF6-mediated K63 ubiquitination of STAT3 to enhanced kinase activation, direct causal evidence at the structural level remains to be established. Ongoing and future studies will therefore focus on (1) mapping the precise lysine residue(s) on STAT3 targeted by TRAF6-dependent K63 ubiquitination, (2) generating and functionally characterizing ubiquitination-deficient STAT3 mutants in PASMCs, and (3) further delineating the molecular mechanisms by which K63-linked chains promote STAT3 phosphorylation and nuclear activity.

## Data Availability

The original contributions presented in the study are included in the article/Supplementary Material, further inquiries can be directed to the corresponding authors.
