# Supplementary material for: TRAF6 promotes pulmonary hypertension by enhancing K63-linked ubiquitination and activation of STAT3
Source: Front Pharmacol. 2026 Jun 26;17:1850163. doi: 10.3389/fphar.2026.1850163 (PMC13349922; doi:10.3389/fphar.2026.1850163)
Supplement: Supplementary file 1 [file Table1.DOCX]

**Supplementary imformation**

**Table S1. Reagent Sources**

| **Reagent/Resource** | **Source** | **Identifier** |
| --- | --- | --- |
| **Chemicals** |  |  |
| MCT | Sigma-Aldrich | C2401-1G |
| Dulbecco’s modified Eagle’s medium | Gibco | 11965-092 |
| FBS | Gibco | 10099-141 |
| PBS | Servicebio | G4202 |
| Triton X-100 | Beyotime | P0096-100ml |
| RIPA lysis buffer | Beyotime | P0013B |
| PMSF protease inhibitor | Beyotime | ST507 |
| Proteaseand phosphatase inhibitors | Beyotime | P1081 |
| Cycloheximide (CHX) | MCE | HY-12320 |
| MG132 | MCE | HY-13259 |
| Chloroquine (CQ) | MCE | HY-17589A |
| DMSO | Biofroxx | 1084ML500 |
| BeyoMag™ Protein A+G Magnetic Bead | Beyotime | P2108 |
| One Step TUNEL Apoptosis Assay Kit | Beyotime | C1089 |
| BeyoClick™ EdU-488 Cell Proliferation Detection Kit | Beyotime | C0071S |
| Cell Counting Kit-8 | Beyotime | C0037 |
| Rat IL-6 Recombinant protein | PeproTech | 400-06-10UG |
| 3-Color Prestained Protein Ladder | YaZyme | WJ103 |
| **Antibodies** |  |  |
| STAT3 | CST | 12640S |
| p-STAT3 | CST | 9145S |
| TRAF6 | CST | 67591S |
| β-actin | Proteintech | 66009-1-Ig |
| Cleaved Caspase-3 | CST | 9661S |
| HA-Tag | CST | 3724T |
| Anti-DYKDDDDK Tag | Proteintech | 66008-4-Ig |
| Ki67 | Proteintech | 28074-1-AP |
| HRP goat anti mouse | Beyotime | A0216 |
| HRP goat anti rabbit | Beyotime | A0208 |
| Normal Rabbit IgG | Beyotime | A7016 |
| Alexa Fluor® 488荧光Anti-alpha smooth muscle Actin抗体 | Abcam | Ab184675 |
| Goat Anti-Mouse IgG H&L (Alexa Fluor® 488) | Abcam | Ab150113 |
| Donkey Anti-Rabbit IgG H&L (Alexa Fluor® 647) | Abcam | Ab150075 |

**Table S2. Sequences of Small Interfering RNAs (siRNAs) Targeting Rat TRAF6**

| Primer name | Sequence from5ʹ to 3ʹ |
| --- | --- |
| siTRAF6-1(sense) | GGUAAAGUAUCCAAAUAAdTdT |
| siTRAF6-1(antisense) | UUUAUUUGGACACUUUACCdTdT |
| siTRAF6-2(sense) | CGAGGAUCAUCAAGUACAUdTdT |
| siTRAF6-2(antisense) | AUGUCAUUGAUGAUCCUCGdTdT |

**Table S3. Primers used for qPCR**

| **Oligonucleotide** | **Sequence from 5ʹ to 3ʹ** |
| --- | --- |
| *Stat3* -F | TCGTTCTGGGTCTGGCTAGA |
| *Stat3*-R | GGGGGCTTTGTGCTTAGGAT |
| *Traf6-F:* | GCGCCTAGTAAGACAGGACC |
| *Traf6-R:* | TGCTTCCATCTCGGCAACTT |
| *ACTB-F* | AACACAGTGCTGTCTGGTG |
| *ACTB-R* | GTAACAGTCCGCCTAGAAGC |

**Table S4. AAV vectors used in this study**

The recombinant AAV vector was purchased from PackGene Biotech (Guangzhou, China). This is a self-complementary AAV vector of undefined serotype carrying an enhanced SM22α promoter driving simultaneous expression of EGFP, RTraf6, and a miR-30-framed shRNA, followed by WPRE3 and SV40 polyA signal, flanked by AAV2 ITRs. The vector was supplied at a genomic titer of 3.57×10^12^ GC/mL (as determined by qPCR targeting the ITR sequence with primers FWD ITR: 5'-GGAACCCCTAGTGATGGAGTT-3' and REV ITR: 5'-CGGCCTCAGTGAGCGA-3'). After 20-fold calibration, the delivery titer was reported as 1×10^13^ GC/mL. Vector purity showed no visible contaminating bands by SDS-PAGE silver staining. Endotoxin levels were <10 EU/mL as determined by the LAL assay.

| Vector name | ssAAV-mEnSM22α-EGFP-RTraf6-miR30shRNA-WPRE3-SV40pA |
| --- | --- |
| Serotype | Not specified |
| Promoter | mEnSM22α (enhanced SM22α) |
| Transgene(s) | EGFP, RTraf6, miR-30-based shRNA |
| Titer (GC/mL) | 3.57 × 10¹² (qPCR); 1 × 10¹³ (calibrated) |
| Dose administered | 1-5 × 10¹¹ GC per animal |
| Administration route | Intratracheal instillation |
| Supplier | PackGene Biotech (Guangzhou, China) |

**Figure. S1** Characterization of Primary pulmonary arterial smooth muscle cells.

PASMCs were isolated from rat pulmonary arteries using the tissue explant culture method and identified by immunofluorescence staining. Cells exhibited characteristic spindle-shaped morphology with positive staining for α-smooth muscle actin (α-SMA, green fluorescence, FITC-conjugated). Parallel actin stress fibers were clearly visible within the cytoplasm. Scale bar=20 µm.


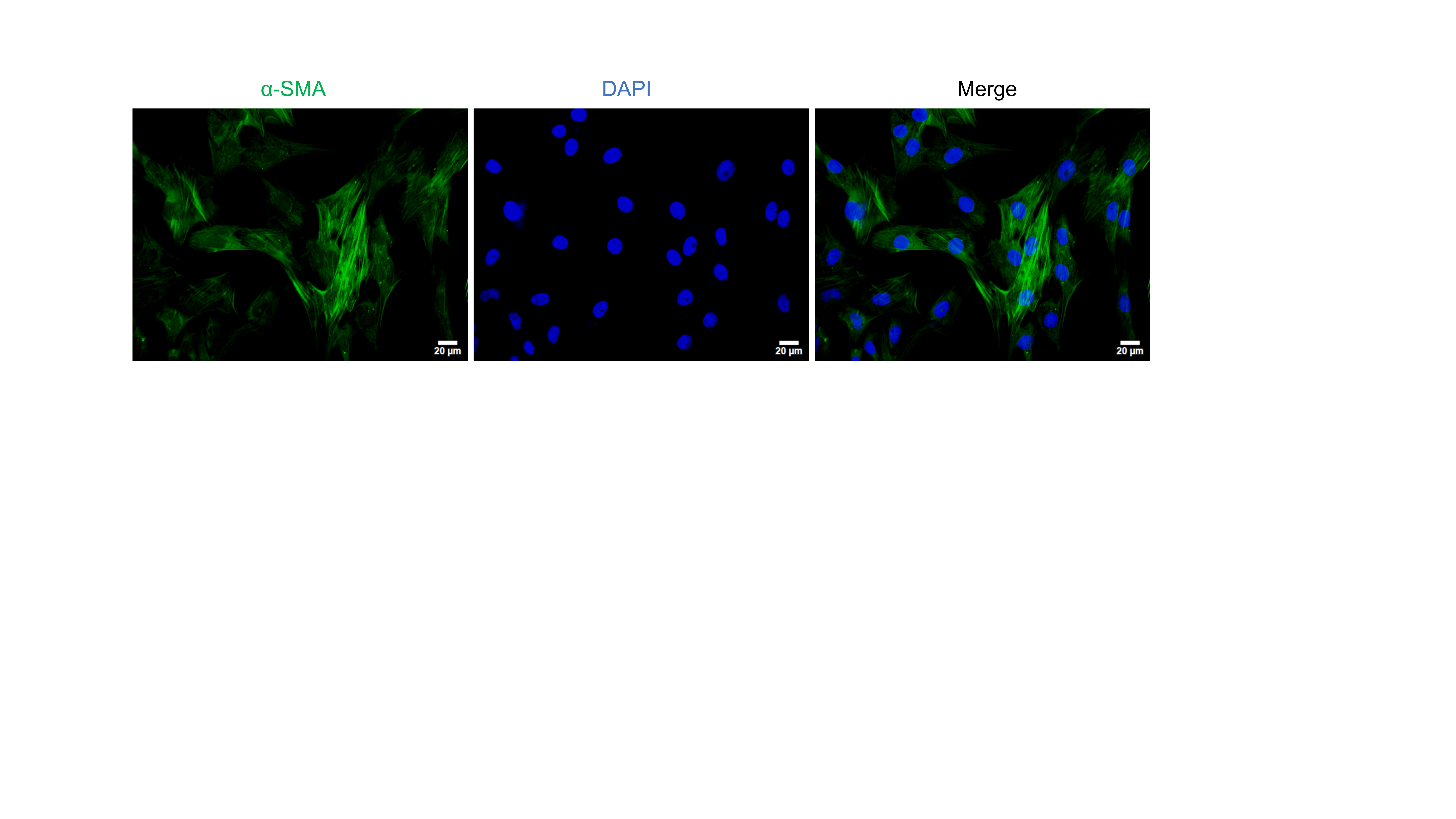


Fig. S1
